# Moderate Aerobic Exercise Regulates Follicular Dysfunction by Initiating Brain-Derived Neurotrophic Factor (BDNF)-Mediated Anti-Apoptotic Signaling Pathways in Polycystic Ovary Syndrome

**DOI:** 10.3390/jcm11195584

**Published:** 2022-09-23

**Authors:** Yaling Zhang, Dejian Chen, Daojuan Wang, Lei Wang, Yajing Weng, Hongwei Wang, Xiaoke Wu, Yong Wang

**Affiliations:** 1 School of Medicine, Jiaxing University, Jiaxing 314001, China; 2 State Key Laboratory of Analytical Chemistry for Life Science & Jiangsu Key Laboratory of Molecular Medicine, Medical School, Nanjing University, Nanjing 210093, China; 3 Department of Anesthesiology and Pain Research Center, The First Hospital of Jiaxing or The Affiliated Hospital of Jiaxing University, Jiaxing 314001, China; 4 Department of Pain, The Affiliated Nanjing Drum Tower Hospital, Medical School, Nanjing University, Nanjing 210008, China; 5 Department of Clinical Laboratory, Affiliated Hospital of Integrated Traditional Chinese and Western Medicine, Nanjing University of Chinese Medicine, Jiangsu Province Academy of Traditional Chinese Medicine, Nanjing 210028, China; 6 Department of Obstetrics and Gynecology, First Affiliated Hospital, Heilongjiang University of Chinese Medicine, Harbin 150040, China

**Keywords:** aerobic exercise, brain-derived neurotrophic factor, apoptosis, neuroendocrine, polycystic ovary syndrome

## Abstract

Polycystic ovary syndrome (PCOS) is a common endocrine disorder among women. Moderate aerobic exercise intervention is considered an initial treatment strategy for managing PCOS. Brain-derived neurotrophic factor (BDNF) is an important molecular mediator and a beneficial response to exercise. We aimed to investigate the expression pattern and underlying molecular mechanisms of this neurotrophic factor during follicle development in ovarian tissues. The PCOS model was established by subcutaneous injection of 60 mg/kg dehydroepiandrosterone (DHEA) into the neck of Sprague Dawley rats for 35 consecutive days. PCOS rats then received aerobic exercise for 8 weeks. Body/ovarian weight and peripheral serum hormone levels were observed. Immunohistochemistry combined with Western blot analysis and fluorescence quantitative polymerase chain reaction were used to detect the changes in BDNF-TrkB/p75NTR pathway, apoptosis, and inflammatory factors. We show that moderate aerobic exercise not only reverses the PCOS phenotype but also activates the BDNF-TrkB pathway and initiates downstream targets. p-TrkB upregulates and phosphorylates phosphatidylinositol 3-kinase (PI3K) and protein kinase B (Akt) to inhibit apoptosis. In addition, aerobic exercise therapy reduces the high expression of p75NTR in the ovarian tissue of PCOS rats and initiates the anti-apoptotic effect from the downstream pathway of NF-κB/JNK. Our in vitro results state that treatment with BDNF ameliorated dihydrotestosterone (DHT)-induced granulosa cells (GCs) apoptosis by provoking p-TrkB activation and upregulating the PI3K/AKT pathway. The present study suggests that moderate aerobic exercise regulates follicular dysfunction in PCOS-like rats. One possible mechanism is to initiate the BDNF-mediated anti-apoptotic signaling pathway.

## 1. Introduction

Polycystic ovary syndrome (PCOS) is a common endocrine disorder among women, mainly characterized by hyperandrogenemia, hyperinsulinemia, and chronic anovulation [1,2]. Because of the complex pathogenesis of PCOS, no mechanism-based treatments have been presented against it. However, previous studies support that elevated androgen levels contribute to PCOS [3]. Previous studies show that hyperandrogenism has induced oxidative stress (OS), ovarian fibrosis, chronic low-grade inflammation, mitochondrial dysfunction, and excessive endoplasmic reticulum (ER) stress in the ovary that ultimately affected follicle development [4,5,6,7,8]. Recent evidence has revealed that the pathophysiological mechanisms of PCOS are associated with neuroendocrine impairments [9,10,11]. Thus, increased androgen signaling in the brain and ovary may be a potential mechanism in the pathophysiology of PCOS.

Regular physical activity can prevent or reduce the risk of many diseases [12]. Moderate aerobic exercise is the preferred treatment for PCOS. Exercise can reduce androgen and insulin levels in PCOS, improve the endocrine environment of the ovary, and play an important role in promoting ovulation [13,14]. Our previous research has shown that aerobic exercise can alleviate hyperandrogenism-induced ER stress and reverse ovarian granulosa cells (GCs) apoptosis in PCOS-like rats [15]. In addition, exercise stimulates the release of neurotransmitters and neurotrophins such as nerve growth factor (NGF), brain-derived neurotrophic factor (BDNF), neurotrophin-3, and neurotrophin-4/5 in an activity-dependent manner [16,17]. They play an important role in regulating neuronal survival and differentiation and in non-neuronal tissues such as ovarian follicles [18]. Some studies have shown that endometriosis is associated with low follicular-fluid BDNF levels, and diminished ovarian reserve is associated with increased follicular-fluid NGF levels [19]. In vitro treatment with an appropriate concentration of BDNF can promote oocyte maturation and embryonic development [20]. In addition, BDNF affects the development of the materno-fetal-placental unit in terms of differentiation, proliferation, and placental nutrient transport [21]. However, it has also been reported in some studies that the BDNF levels of PCOS patients in plasma and in follicular fluid were higher than values obtained in healthy controls [22]. Subsequent findings suggest that chronic low-dose inflammation in PCOS may interact with BDNF to contribute to the development of depression [23]. In conclusion, BDNF signaling events have substantial roles in the ovary, and BDNF expression and levels have been linked with follicle organization during ovarian development, follicle recruitment, and growth and oocyte maturation [24]. However, BDNF expression patterns in non-neuronal tissues, such as the ovary, and the underlying mechanism of oocyte maturation remain unclear.

BDNF binds to the high-affinity tropomyosin-related kinase receptor type B (TrkB) and exerts its pro-survival effects by activating the downstream signaling pathways, including the phosphatidylinositol 3-kinase (PI3K)-Akt pathway [25]. BDNF can also activate the pan-neurotrophin low-affinity co-receptor p75 (p75NTR) [26,27,28]. In many species, BDNF can affect oocyte maturation and early embryo development [29]. However, the role of BDNF and its receptor in PCOS remains to be determined. In addition, the role of BDNF-TrkB/p75NTR signaling in follicular development is limited and incompletely understood. In particular, exercise has been shown to promote the expression of BDNF in hippocampus and cerebral cortex [30]. Whether exercise can directly affect the expression of BDNF and its receptors in ovarian tissues remains to be further explored. In this study, we explored the potential mechanism by which moderate aerobic exercise may modulate follicular dysfunction by increasing the expression of BDNF in PCOS. As our understanding of the expression and underlying molecular mechanisms of these neurotrophic factors in the human ovary grows, new diagnostic and therapeutic applications for the management of patients with infertility and ovarian pathology, as well as improvement in oocyte quality, will be developed.

## 2. Subjects and Methods

### 2.1. Animal Models and Groups

The Shanghai Xipuer-Bikai Laboratory Animal Co., Ltd. (Shanghai, China) provided 60 specific pathogen-free (SPF) Sprague Dawley^®^ (SD) female rats (21 days old, 50–60 g). All animal experiments were performed in accordance with the guidelines of the Institutional Animal Care and Use Committee (IACUC) and approved by the Institutional Research Animal Committee of Nanjing University. The rats were housed in closed cages under controlled temperature (22 ± 2 °C) and light (12:12 h, light:dark). All SD rats were initially divided into three groups: the control group (no treatment, n = 10), model group (DHEA treatment, n = 10), and exercise group (DHEA treatment + treadmill training treatment, n = 10). The PCOS rats were induced with dehydroepiandrosterone (DHEA) (6 mg/100 g BW) for 35 consecutive days.

### 2.2. Implementation of Moderate Aerobic Exercise

After the PCOS model was established by DHEA subcutaneous injection, treadmill aerobic training was conducted at 10:00 a.m. the next day. In the first week, adaptive treadmill training was performed. Rats were trained at 5 m/min–10 m/min for 10 min on the first day, and training length increased by 30 min per day, ending at up to 15 m/min for 60 min on the sixth day. Then, seven-week regular exercise training was performed at 15 m/min for 60 min per day (5 m/min for the first 5 min, 10 m/min for 10 min, and 15 m/min for the remaining 45 min), six days per week for eight weeks. Furthermore, at the end of the 8-weeks treatment period, blood samples and tissues were collected immediately.

### 2.3. Isolation and Culture of Granulosa Cells (GCs)

In order to enhance multiple follicular development, 23-day-old immature female rats were injected with pregnant mare serum gonadotropin (PMSG) (20 IU) 48 h in advance. The rats were killed by cervical dislocation and were disinfected with 75% alcohol for 20 min. The ovaries were quickly removed on a super-clean bench, and the follicles were punctured with microscopic tweezers to release GCs. Cell debris was removed using 100 μm cell strainers. The primary GCs was cultured in DMEM-F12 containing 10% fetal bovine serum (FBS, Gibco, New York, NY, USA) and 1% penicillin-streptomycin solution (Gibco, New York, NY, USA), in a cell incubator at 37 °C (95% relative humidity, 5% CO_2_). Cell growth was monitored intermittently.

### 2.4. CCK8 Assay

GCs were seeded in 96-well plates (1 × 10^5^ cells/well) for 48 h and then treated with various concentrations of dihydrotestosterone (DHT) and BDNF (Cayman Chemical, Ann Arbor, MI, USA) for the indicated times. Cell counting kit-8 (CCK-8) solution (10 μL; A311-02-AA, Vazyme Biotech, Nanjing, China) was added to each well, followed by incubation for 4 h at 37 °C. The absorbance at 450 nm was measured using a microplate reader.

### 2.5. Cell Apoptosis Assessment

Cell apoptosis was assessed using an Alexa Fluor 488-conjugated annexin V and propidium iodide (PI) (C1062L, Beyotime, Shanghai, China) detection kit. Primary GCs were treated with or without DHT, BDNF, and ANA-12, and then, 5 μL of PI and 10 μL of annexin V–FITC was added to the cells. FITC–Annexin V-positive cells were considered apoptotic cells.

### 2.6. Hematoxylin and Eosin (H&E) Staining

Paraffin slides were stained with hematoxylin and eosin to examine the pathological structural alterations of the rat ovary and hippocampus under an optical microscope (Leica Microsystems, Weztlar, Germany).

### 2.7. Serum Hormone Measurement

The rats were anesthetized with 1% pentobarbital sodium (40 mg/kg, ip), and blood was drawn from the superior vena cava. The serum was separated immediately and stored at −80 °C for further determination of testosterone (T), luteinizing hormone (LH), and follicle-stimulating hormone (FSH) levels through enzyme-linked immunosorbent assay (ELISA) (Elabscience Biotechnology, Wuhan, China).

### 2.8. Nissl Staining

Nissl staining was performed to assess neuronal survival. The sections were rinsed in deionized water, dipped in a warm (50 °C) solution of 1% thionine for 45 min, and differentiated with 70% alcohol for approximately 5 min.

### 2.9. ELISA

Serum was collected for detecting IL-1β using ELISA kits (Elabscience Biotechnology). The ELISA provided a detection range ranging from 31.25 to 2000 pg/mL, and the sensitivity of the ELISA kit was 18.75 pg/mL. According to the manufacturer’s instructions, 450 nm was regarded as the most suitable wavelength for measuring absorbance.

### 2.10. Immunohistochemistry (IHC) and Immunofluorescence (IF)

After antigen retrieval, dewaxed and rehydrated sections were treated with 3% hydrogen peroxide and then 5% BSA and incubated overnight at 4 °C with primary antibody BDNF (1:100, Wanleibio, Shenyang, China). Slides were then incubated with a secondary goat anti-rabbit IgG horseradish peroxidase (HRP) at 37 °C for 30 min. Sections were consequently stained with diaminobenzidine for 10 min, counterstained with hematoxylin (Beyotime, Shanghai, China), covered with coverslips, and observed under an optical microscope. The histology was quantified with Image Pro Plus 6.0 based on optical density.

The tissue steps and cell climbing pieces before the sections were incubated with the primary antibody were the same for immunohistochemical staining. Sections were incubated overnight at 4 °C with antibodies against BDNF (1:100, Wanleibio), TrkB (1:100, Wanleibio), p75NTR (1:100, 55014-1-AP, Proteintech, Chicago, IL, USA), and Cleaved-Caspase-3 (1:100, 66470-2-Ig, Proteintech) at a 1:100 dilution. Fluorescently labeled secondary antibodies were diluted (1:1000) and incubated in the dark at 25 °C for 2 h. Nuclei were counterstained with 4′,6-diamidino-2-phenylindole (C1002, Beyotime) at a dilution of 1:2000 for 30 min. Images were photographed using an Olympus laser scanning confocal microscope (FV3000, Tokyo, Japan). Fluorescence intensity was quantified using Image-Pro Plus 6.0 (Media Cybernetics, Rockville, MD, USA).

## 3. TUNEL Analysis

TUNEL assays were performed to detect Bcl-2 and caspase 3 levels in ovary sections. A Fluorescein (FITC) TUNEL Cell Apoptosis Detection Kit (G1501-100T, Servicebio, Wuhan, China) was used in accordance with the manufacturer’s instructions. Slides treated with DNase I for 30 min served as positive controls. DAPI was used to stain the nuclei.

### 3.1. Quantitative Real-Time PCR (qRT-PCR)

The total RNA was extracted from the GCs in the ovaries with TRIzol reagent (Beyotime), and the cDNA was synthesized with a reverse transcription kit (Vazyme, China). Quantitative RT-PCR was performed with the ABI Viia7 real-time PCR system (ABI, Los Angeles, CA, USA) by using the SYBR™ Green PCR Master Mix (Vazyme). Quantitative RT-PCR was performed as follows: Stage 1, pre-denaturation (Rep: 1, 95 °C, 30 s); Stage 2, circular reaction (Reps: 40, 95 °C, 10 s; 60 °C, 30 s); Stage 3, melting curve (Rep: 1, 95 °C, 10 s; 60 °C, 60 s; 95 °C, 15 s). The primers used in this study are shown in Table 1. The critical threshold cycle (Ct) value was determined for each reaction for relative quantification using the 2^−∆∆Ct^ method. Glyceraldehyde 3-phosphate dehydrogenase (*GAPDH*) was used as an internal control.

### 3.2. Western Blot

The tissue samples were homogenized through mechanical disruption in RIPA lysis buffer (P0013B, Beyotime) containing 1 mM Pierce^TM^ phosphatase inhibitor (B15001, Selleck, TX, USA) and 0.1% Halt^TM^ protease inhibitor cocktail (B14001, Selleck). The gel electrophoresis system of Bio-Rad (12% SDS polyacrylamide) was used to separate proteins from samples that contained the same protein quantity (30 µg); the proteins were then transferred onto the polyvinylidene difluoride membranes (IPVH00010, Merck Millipore, Burlington, MA, USA). Target bands were incubated at 4 °C overnight with corresponding primary antibodies against BDNF (1:500, WL0168, Wanleibio, Wuhan, China), TrkB (1:500, WL00839, Wanleibio), p-TrkB (1:500, WL02988, Wanleibio), p75NTR (1:500, 55014-1-AP, Proteintech, Chicago, IL, USA), PI3K (1:1000, 4292S, CST, Boston, MA, USA), p-PI3K (1:1000, AF3242, Affinity, Boston, MA, USA), AKT (1:1000, 4691T, CST), p-AKT (1:1000, 4060T, CST), NF-κB (1:1000, 8242S, CST), p-NF-κB (1:1000, 3033S, CST), JNK (1:1000, 9252T, CST), p-JNK (1:1000, 4668S, CST), IL-1β (1:1000, ab9722, Abcam, Cambridge, UK), IL-6 (1:1000, 21865-1-AP, Proteintech), Bcl-2 (1:500, Wanleibio), Bax (1:1000, 50599-2-Ig, Proteintech), cleaved caspase-3 (1:1000, 66470-2-Ig), AR (1:1000, ab52615, Abcam), FSHR (1:1000, BS5724, Bioworld, Nanjing, China), Cyp11α1 (1:1000, BS6578, Bioworld), Cyp19α1 (1:1000, BS6580, Bioworld), and GAPDH (1:5000, Bioworld), followed by the addition of HRP-labeled secondary antibodies. The blots were visualized using chemiluminescent detection (Merck Millipore, NJ, USA). Densitometric analysis was performed with Image J.

### 3.3. Statistical Analysis

Values were expressed as the mean ± SEM. All statistical analyses were performed with GraphPad (Prism 7.0). Multiple comparisons were implemented through one- and two-way ANOVA followed by Tukey’s post-hoc test. Binary variables were compared using a *t*-test, and a *p*-value < 0.05 was regarded as statistically significant.

## 4. Results

### 4.1. Effects of Moderate Aerobic Exercise on Ovarian Dysfunction in DHEA-Induced PCOS Rats

**Figure 1:**
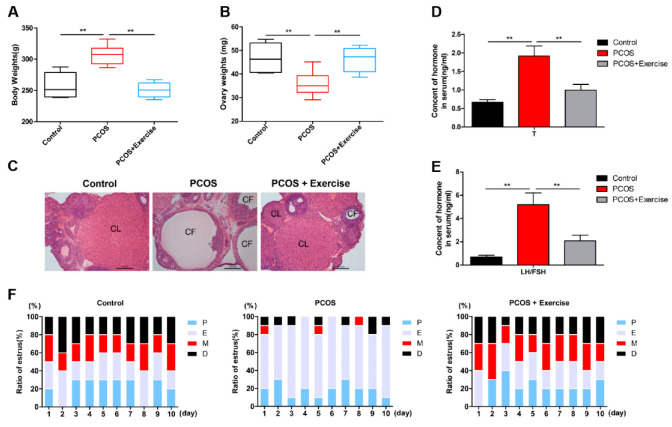
Aerobic exercise reversed the PCOS phenotype. Rats received DHEA for the induction of polycystic ovarian syndrome, together with or without exercise treatment. (**A**) Rat body weights were measured in each group after 8 weeks of exercise treatment. (**B**) Average weight of both ovaries was measured. (**C**) Ovarian and follicular morphology was assessed by H&E staining (10×). (**D**,**E**) Serum T and LH/FSH levels were analyzed using enzyme-linked immunosorbent assay kits. (**F**) The estrus cycle of all rats in the experimental group was continuously shown. n = 10 in each group. Data are shown as mean ± SEM. ** *p* < 0.05. CL, corpus luteum; CF, cystic follicles; P, proestrus; E, estrus; M, metestrus; D, diestrus.

**Figure 2:**
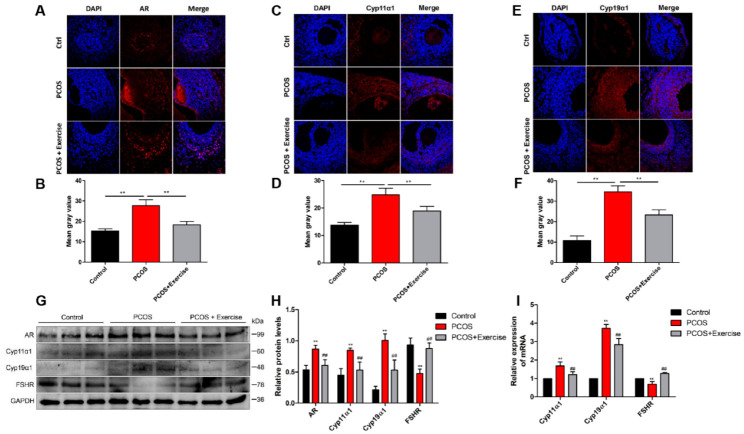
Ovarian dysfunction is improved after treatment with aerobic exercise in DHEA-induced PCOS rats. Rats received DHEA for the induction of polycystic ovarian syndrome, together with or without exercise treatment. (**A**–**E**) The levels of AR, Cyp11α1, and Cyp19α1 were measured with immunohistochemical staining. (**B**–**F**) These bar charts show the quantitative analysis. (**G**,**H**) The expression of AR, Cyp11α1, and Cyp19α1 in ovaries was assessed by Western blot assay. (**I**) mRNA expression of AR, Cyp11α1, and Cyp19α1 factors was analyzed by real-time PCR. n = 10 in each group. Data are shown as mean ± SEM. ** *p* < 0.05, vs. control group; ## *p* < 0.01, vs. PCOS group.

In this study, the bodyweight of rats induced with DHEA was higher than that in the control group, but after 8 weeks of aerobic (treadmill) exercise, their weight was significantly reduced (Figure 1A). The ovarian weight of PCOS rats also markedly increased after the exercise treatment (Figure 1B). In addition, compared to the control group, the ovaries in PCOS rats had more atypical follicles but almost no corpus luteum. However, multiple immature follicles were substantially reduced after exercise treatment, whereas the number of corpus luteum evidently increased (Figure 1C). Furthermore, the elevated T and LH/FSH levels in PCOS rats declined to normal levels after the exercise treatment (Figure 1D,E). The estrus cycle of all rats was continuously observed for two cycles (10 days). The results show that the rats in the PCOS group lost their regular estrus cycle. After exercise intervention, the estrus cycle returned to normal (Figure 1F). Notable changes were also observed in genes related to follicular development after 8 weeks. AR, Cyp11α1, and Cyp19α1 levels increased, while FSHR levels decreased in the ovaries of PCOS rats (Figure 2A,I). In addition, fasting blood sugar (FBG) was measured using a glucometer, and compared with the control group, the FBG levels in the PCOS group were increased. Relative to the PCOS group, the PCOS + exercise group had decreased FBG levels. However, there was no statistical difference (Table 2).

### 4.2. Location and Expression of BDNF in the Hippocampus of PCOS Rats after Moderate Aerobic Exercise

The effects of exercise in the brain are most apparent in the hippocampus [31], and thus, H&E and Nissl staining were conducted to explore the effects of aerobic treadmill exercise in the hippocampus of DHEA-induced PCOS rats. Compared with the control group, Nissl stains in the hippocampal CA1, CA3, and DG regions of PCOS rats were lighter. After treadmill running, neurons were orderly and densely arranged in the hippocampal regions, and the number of neurons in hippocampal DG and CA3 areas was notably enhanced, similar to the hippocampal regions of the control group (Figure S1A,B).

Analysis of immunofluorescence data showed that the BDNF level in the PCOS group was significantly lower than that in the other groups. A significant rise in BDNF in the PCOS group was observed after 8 weeks of exercising (Figure S1C,D). Western blot analysis showed that the expression of BDNF, TrkB, and p-TrkB proteins decreased and p75NTR proteins increased in the hippocampus of the PCOS group compared to the control group. After the treadmill exercise, the above phenomenon was corrected (Figure S1E,F).

### 4.3. Location and Expression of BDNF, TrkB, and p75NTR in Ovarian Follicles of PCOS Rats after Moderate Aerobic Exercise

**Figure 3:**
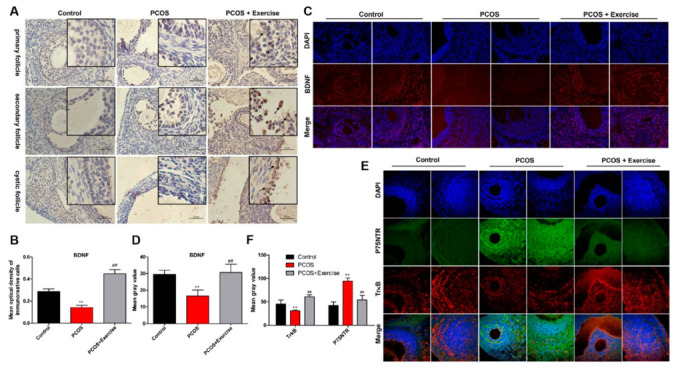
The expression of BDNF, TrkB, and p75NTR in ovarian follicles of PCOS rats after aerobic exercise. Rats received DHEA for the induction of polycystic ovarian syndrome, together with or without exercise treatment. (**A**,**B**) The expression levels of BDNF in ovarian follicles at different stages were analyzed by immunohistochemistry. (**C**,**D**) The expression levels of BDNF in ovarian follicles were analyzed by immunofluorescence. (**E**,**F**) The co-expression of TrkB and p75NTR in ovarian follicles at different stages was detected by immunofluorescence double labeling. n = 10 in each group. Data are shown as mean ± SEM. ** *p* < 0.05, vs. control group; ## *p* < 0.01, vs. PCOS group.

BDNF and its receptors are thought to be key regulatory proteins in the development of the ovary and ovarian regulation in a follicle-stage-dependent manner [32,33]. To detect the location and expression of BDNF, TrkB, and p75NTR in the ovary, immunohistochemistry and double-labeling immunofluorescence were performed. BDNF could be detected in granulosa and membranous cell layers in the ovarian follicle. However, the expression levels of BDNF and TrkB in the PCOS group were lower than those in the control group, but the expression levels of p75NTR were higher. After 8 weeks of aerobic exercise, the PCOS group had substantially enhanced BDNF and TrkB expression, but with decreased levels of p75NTR (Figure 3A–E).

### 4.4. Activated BDNF Signaling in the Ovary of DHEA-Induced PCOS Rats

The expression levels of p75NTR, TrkB, and BDNF can be modulated with response to stimuli through lowering p75NTR or ameliorating BDNF-TrkB signaling [28]. To further confirm that aerobic exercise could activate BDNF signaling in the ovary of DHEA-induced PCOS rats, we analyzed the mRNA and protein expression levels of BDNF, TrkB, and p75NTR in the ovarian tissue from different groups. Based on RT-qPCR and Western blot analysis, the expression level of BDNF in the PCOS group was lower than that in the other groups. Moreover, the expression pattern of TrkB was similar to BDNF, but that of p75NTR was higher in the PCOS group after 8 weeks of aerobic exercise.

**Figure 4:**
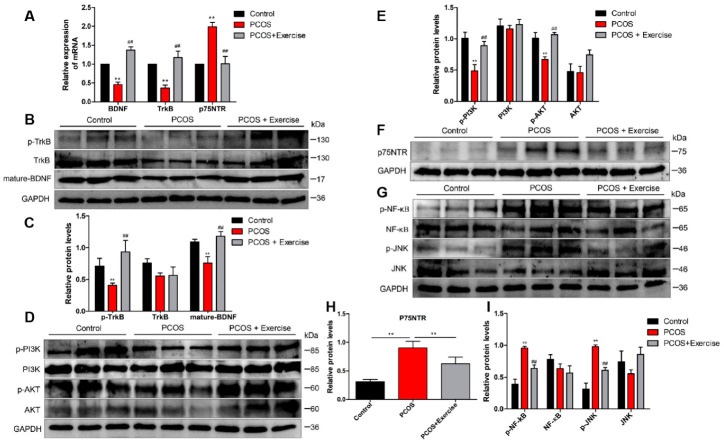
Aerobic exercise activated BDNF signaling in the ovary of DHEA-induced PCOS rats. Rats received DHEA for the induction of polycystic ovarian syndrome, together with or without exercise treatment. (**A**) mRNA expression of BDNF, TrkB, and p75NTR factors in ovarian tissue was analyzed by real-time PCR. (**B**,**C**) The expression of BDNF, TrkB, and p75NTR in ovarian tissue was assessed by Western blot assay. (**D**,**E**) The expression of PI3K, AKT, and p-AKT in ovarian tissue was assessed by Western blot assay. (**F**–**I**) The expression of p75NTR, NF-κB, p-NF-κB, JNK, and p-JNK in ovarian tissue was assessed by Western blot assay. n = 10 in each group. Data are shown as mean ± SEM. ** *p* < 0.05, vs. control group; ## *p* < 0.01, vs. PCOS group.

BDNF expression was substantially enhanced, accompanied by the upregulated phosphorylation of TrkB (p-TrkB) (Figure 4A–C). In addition, the level of p75NTR significantly decreased in rats after exercising (Figure 4F,H).

TrkB and p75NTR are known to engage in disparate signaling pathways downstream of ligand activation. TrkB promotes PI3K/AKT, while p75NTR stimulates NF-κB and JNK pathways. Consistently, Western blot analysis confirmed that PI3K and p-AKT markedly decreased in the PCOS group after 8 weeks of aerobic exercise, and the expression levels of PI3K and p-AKT in the ovarian tissue of rats were significantly upregulated (Figure 4D,E). Furthermore, both NF-κB and JNK pathways were activated in DHEA-induced rats. Treatment with aerobic exercise significantly reduced the expression of phosphorylated protein levels in NF-κB (p-NF-κB) and JNK (p-JNK) pathways (Figure 4G,I).

### 4.5. Moderate Aerobic Exercise Reduced Apoptosis and Inflammation of Ovarian Tissue in PCOS Rats

**Figure 5:**
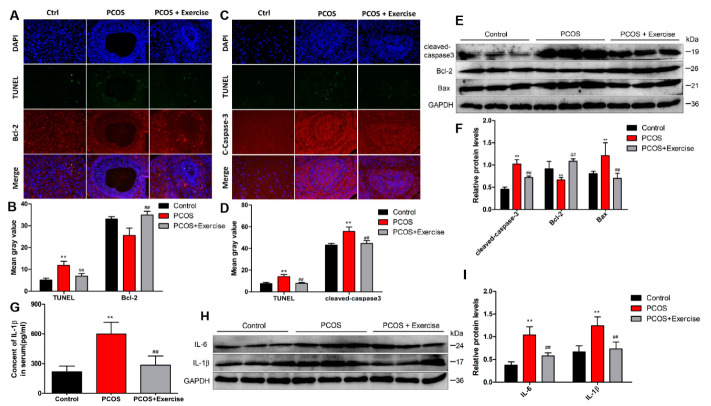
Aerobic exercise reduced DHEA-induced apoptosis and inflammatory response in PCOS rats. Rats received DHEA for the induction of polycystic ovarian syndrome, together with or without exercise treatment. (**A**–**D**) Fluorescence co-staining results of TUNEL Caspase3 and Bcl-2 in ovarian tissues of rats in each group. (**E**,**F**) The expression of cleaved caspase-3, Bax, and Bcl-2 in ovarian tissue of each group by Western blot. (**G**) Quantification of ELISA demonstrated the relative content level of IL-1β in each group. (**H**,**I**) The expression of inflammatory factors IL-6 and IL-1β in ovarian tissue of each group by Western blot. n = 10 in each group. Data are shown as mean ± SEM. ** *p* < 0.05, vs. control group; ## *p* < 0.01, vs. PCOS group.

A TUNEL assay was performed to examine the apoptosis of ovarian tissues in each group. The number of TUNEL-positive cells was significantly higher in the PCOS group than in the control group, but the PCOS + exercise group had fewer TUNEL-positive cells than the PCOS group. In addition, double immunofluorescence staining showed that aerobic exercise increased the expression of anti-apoptotic protein Bcl-2 and decreased the level of cleaved caspase-3 in ovarian tissue of PCOS rats (Figure 5A,D). Consistently, Western blot results demonstrate that hyperandrogenism significantly increased levels of cleaved caspase-3 and Bax and decreased the anti-apoptotic protein Bcl-2 in ovarian tissues compared to the control group. However, levels of cleaved caspase-3 and Bax in ovarian tissues of rats treated with aerobic exercise were remarkably reduced, while the level of Bcl-2 increased (Figure 5E,F). We observed greater production of IL-1β in serum samples of each group. The concentration of serum IL-1β (pg/mL) in the PCOS group was greater than that in the control group. After exercise therapy, the serum IL-1β level was decreased significantly in the PCOS + exercise group (Figure 5G). Moreover, the protein levels of inflammatory cytokines (IL-10, IL-6, IL-1β) in ovarian tissue significantly decreased after aerobic exercise therapy (Figure 5H,I). Our findings indicate that aerobic exercise therapy is capable of suppressing DHEA-induced apoptosis and inflammation in the PCOS model.

### 4.6. BDNF Possibly Alleviates DHT-Induced GC Apoptosis by Upregulating the PI3K/AKT Pathway

Our previous research showed that hyperandrogenism can induce ovarian GCs pyroptosis [7]. Our data showed that BDNF in PCOS rats increased significantly after exercise. Thus, GCs were treated with 5 μM DHT and various BDNF concentrations (0, 10, 100, 1000 ng/mL) for 48 h, and GCs viability after BDNF treatment was analyzed using CCK-8. As expected, the cell viability was rescued after BDNF treatment (Figure S2A,B). To test this hypothesis of BDNF/TrkB signaling, we used the inhibitor for TrkB receptor ANA-12. Results from immunofluorescence and Western blotting assays show that the provoking action of BDNF was abolished in the presence of ANA-12 with diminished expression of p-TrkB compared with DHT + BDNF group (Figure S2C–F).

To further confirm the anti-apoptotic effect of BDNF is dependent on the activation of the PI3K/AKT pathway, we used PI3K inhibitor LY294002 in in vitro studies. The apoptosis rate of GCs was also analyzed by PI and FITC-Annexin V staining.

**Figure 6:**
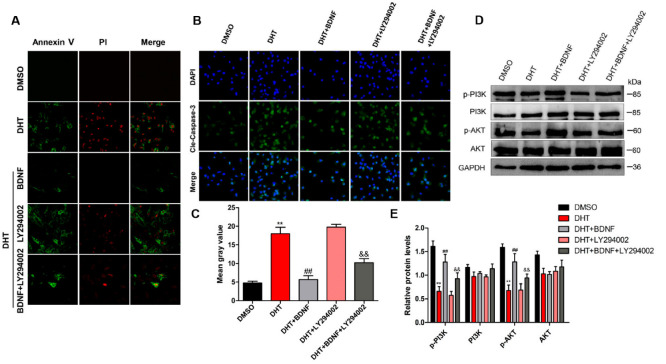
BDNF possibly alleviates DHT-induced GCs apoptosis by upregulating the PI3K/AKT pathway. DHT-induced GCs were treated with BDNF and LY294002 with or without DHT. (**A**) GCs were incubated with annexin V-FITC and PI. The cells were imaged for apoptosis detection using a FV3000 Olympus microscope. (**B**,**C**) Apoptosis protein expression of cleaved caspase-3 was analyzed and quantified using Image-J software (60×). (**D**,**E**) The expression levels of PI3K, p-PI3K, AKT, and p-AKT were detected by Western blot assay. Data are shown as mean ± SEM. ** *p* < 0.05, vs. DMSO group; ## *p* < 0.01, vs. DHT group; && *p* < 0.01, vs. DHT + BDNF group.

As expected, BDNF treatment reversed DHT-induced apoptosis of GCs, and PI3K inhibitor LY294002 eliminated the anti-apoptotic effect of BDNF (Figure 6A). Moreover, BDNF treatment exerted an anti-apoptotic effect on DHT-induced GCs, and cleaved caspase-3 level was decreased using immunofluorescence assay (Figure 6B,C). In addition, Western blotting analysis showed that BDNF treatment caused increased expression of p-PI3K and p-AKT, but LY294002 inhibits their expression in GCs (Figure 6D,E).

## 5. Discussion

Follicular dysplasia in PCOS patients may be caused by abnormal endocrine and paracrine factors and changes in follicular microenvironment. Recently, a growing number of studies have shown that neurotrophins and their receptors are also expressed throughout the reproductive system [34]. BDNF is a neurotrophic protein first discovered in the pig brain by scientists in 1982. It is mainly expressed in the central nervous system, with the highest content in the hippocampus and cortex. Studies on the role of BDNF and its receptor in the ovaries of humans and other mammals are increasing. It is inferred that BDNF may be a physiological regulator promoting follicle development, granulosa cell proliferation, and oocyte maturation [24,35]. BDNF is typically synthesized as a large precursor protein (pro-BDNF), which exhibits exclusive binding to p75NTR. Pro-BDNF can be cleaved to form mature BDNF (m-BDNF), and both are biologically active. Mature BDNF signals through its high-affinity receptor TrkB [36,37]. However, the interactions between BDNF-TrkB and pro-BDNF-p75NTR are complex and can be modified at various levels (alternative forms, alternative receptors/signaling pathways). Therefore, the underlying mechanism is not fully understood and should be further explored.

Exercise can enhance the expression of BDNF. Exercise plays an important role in the prevention and treatment of PCOS as a non-drug intervention method. In addition, exercise requires high intensity, duration, and muscle group allocation. Moderate exercises are beneficial for most women and could improve fertility for those with anovulatory disorders such as PCOS [38,39]. There was also a study showing that acute high-intensity intermittent exercise increased serum BDNF concentration and attenuated the emotional states of tension, depression, and anger [40]. The findings suggested exercise as a strategy to attenuate the deleterious sensations occasioned by ovarian hormonal fluctuations and regulate ovarian function. In this study, we first established that DHEA is a major androgen precursor. Hyperandrogenism-induced PCOS rats showed weight gain, ovarian weight loss and polycystic changes, and hormone disturbance. When the rats were subjected to treadmill exercise (6 d/wk, 1 h/d at a pace of 15/min for 8 week), PCOS rats lost body weight, ovarian morphology returned to normal, and hormone disturbance was corrected. The intensity of the exercise was based on References [41,42]. This is consistent with previous studies that showed moderate exercise may help to recover optimal hormonal balance and restore ovulation in overweight and obese women with PCOS [43]. Moderate aerobic exercise can improve follicular dysfunction in PCOS rats, which may be associated with the increased expression of BDNF. Interestingly, we found that BDNF and its receptors TrkB and p75NTR were not only expressed throughout ovarian follicle development but also in the hippocampus after exercise. BDNF is inferred to cross the blood–brain barrier in a bidirectional manner [44,45]. Moreover, the level of circulating BDNF further increased, but in addition to the brain, BDNF was also released in the skeletal muscles, PBMCs, vascular endothelial cells, and platelets [46,47]. Nevertheless, we considered that our study mainly focused on the changes in the PCOS model rats after they received exercise training, rather than explaining the effect of exercise. Although the experimental purpose has been reached, due to the lack of the control + exercise group, the comparison between the changes in the control group after exercise and the changes in the model group after exercise is still inconclusive, which is also the limitation of this study. In addition, the understanding of BDNF is still in its infancy, and further exploration of the candidate sources and release mechanisms of BDNF in exercise is needed. In addition to its well-established neurotrophic action, BDNF also possesses anti-apoptosis, anti-oxidation, and autophagy-suppressing qualities [48,49].

Previous studies have shown that neurotrophic factors directly regulate the function of somatic and granulosa cells in the ovary by binding to corresponding receptors rather than regulating the development of primary to secondary follicles through the ovarian nerves [50,51]. Moreover, studies have shown that BDNF in the ovary is secreted by cumulus and granulosa cells; therefore, the concentration of BDNF must be related to the proliferation of follicular granulosa cells. The BDNF level decreased when granulosa cells were apoptotic [52], and in this study, DHEA-induced PCOS rats showed increased expression of apoptotic proteins (cleaved caspase-3/Bax) and decreased expression of anti-apoptotic B-cell lymphoma 2 (Bcl-2) in the ovary. The results are consistent with previous findings that the beneficial effects of BDNF involve the induction of anti-oxidative thioredoxin, with the resultant expression of Bcl-2. In addition, the number of TUNEL-positive cells was significantly higher in the ovarian tissue of PCOS rats. After 8 weeks of aerobic exercise therapy, the PCOS phenotype was reversed, the expression of apoptotic proteins was downregulated, and the anti-apoptotic protein was upregulated in the ovary. We infer that aerobic exercise increases BDNF, plays an anti-apoptotic role, and corrects ovarian dysfunction. We analyzed the mRNA and protein expression level of BDNF, TrkB, and p75NTR in ovarian tissues and found that the activation of the BDNF-TrkB pathway initiated downstream targets. p-TrkB upregulates and phosphorylates phosphatidylinositol 3-kinase (PI3K) and Akt to inhibit apoptosis. Moderate aerobic exercise therapy may also reduce the high expression of p75NTR in the ovarian tissue of PCOS rats, initiate the anti-apoptotic effect mediated by the downstream pathway of NF-κB/JNK, and thus reduce apoptosis of ovarian oocytes and granulosa cells. In addition, the protein levels of inflammatory cytokines (IL-6 and IL-1β) in ovarian tissue of PCOS rats significantly decreased after aerobic exercise therapy. However, it remains completely unclear whether aerobic exercise benefits BDNF in the process of exerting anti-inflammatory effects. These findings suggest that BDNF could be a candidate neurotrophic factor capable of improving follicular dysfunction.

In conclusion, we show that low levels of BDNF in the ovarian follicle of PCOS rats may be the potential cause of follicular development disorders. Moderate aerobic exercise can enhance the expression of BDNF and initiate the BDNF-mediated anti-apoptotic signaling pathway, thus reversing the ovarian phenotype of PCOS. Exercise plays an important role in the prevention and treatment of PCOS as a non-drug intervention method. However, the limitations of this study included that the exercise protocol was relatively simple, as the rats were only trained on a treadmill and not engaged in other types of aerobic exercise, such as swimming or voluntary wheel running. There is also a lack of studies evaluating exercise in this study. In addition, the temporal and spatial differences in the expression of BDNF and its receptor during ovarian and follicular development suggest that the physiological functions of the BDNF signaling pathway vary in follicles at different developmental stages. Presently, the mechanism for signal transduction of BDNF and its receptors in oocytes, follicular granulosa, and stromal cells is not completely clear. Thus, the expression of BDNF in follicles at various stages, whether as pathogenesis of PCOS or just a biochemical indicator, in the pathophysiological process of PCOS, need to be further studied. From a clinical point of view, due to the high prevalence of PCOS in reproductive-aged women, it is important that future studies about the effectiveness of lifestyle interventions in this PCOS patient population are robustly designed to better provide new ideas for future clinical practice guidelines/recommendations.

## Figures and Tables

**Table 1:** Sequences of primers designed for RT-qPCR.

Genes	Forward	Reverse
*BDNF*	5′-GTGTGACAGTATTAGCGAGTGGG-3′	5′-ACGATTGGGTAGTTCGGCATT-3′
*TrkB*	5′-GGCATCACCAACAGTCAGC-3′	5′-GCATCCTTCAGGGTCTTCA-3′
*p75NTR*	5′-AGGGCACATACTCAGACGAA-3′	5′-AGATGGAGCAATAGACAGGAAT-3′
*FSHR*	5′-CAACCTCCGATATCTGTTAATA-3′	5′-CATTCTTACTCAGCCATACAGT-3′
*Cyp11α1*	5′-GGATGCGTCGATACTCTTCTCA-3′	5′-GGACGATTCGGTCTTTCTTCCA-3′
*Cyp19α1*	5′-AACCCGAGCCTTTGGAGAA-3′	5′-GGCCCGTCAGAGCTTTCA-3′
*GAPDH*	5′-AGGTCGTGTGAGGGATTG-3′	5′-TTAGTAGTAGTAGGGGGGGTCA-3′

**Table 2:** The levels of FBG in three experimental groups.

Group	FBG	*p*
Ctrl	3.76 ± 0.47	-
PCOS	4.68 ± 1.20	0.095
PCOS + Exercise	4.04 ± 0.62	0.253

## Supplementary Materials

The following supporting information can be downloaded at: https://www.mdpi.com/article/10.3390/jcm11195584/s1, Figure S1: Aerobic exercise increased the expression of BDNF and its receptor in the hippocampus; Figure S2: BDNF treatment increased the cell viability of DHT-induced GCs and provoked p-TrkB activation.
