# Supplementary material for: Moderate Aerobic Exercise Regulates Follicular Dysfunction by Initiating Brain-Derived Neurotrophic Factor (BDNF)-Mediated Anti-Apoptotic Signaling Pathways in Polycystic Ovary Syndrome
Source: J Clin Med. 2022 Sep 23;11(19):5584. doi: 10.3390/jcm11195584 (PMC9571561; doi:10.3390/jcm11195584)
Supplement: Supplementary file 1 [file jcm-11-05584-s001.zip › jcm-1875808-supplementary.pdf]

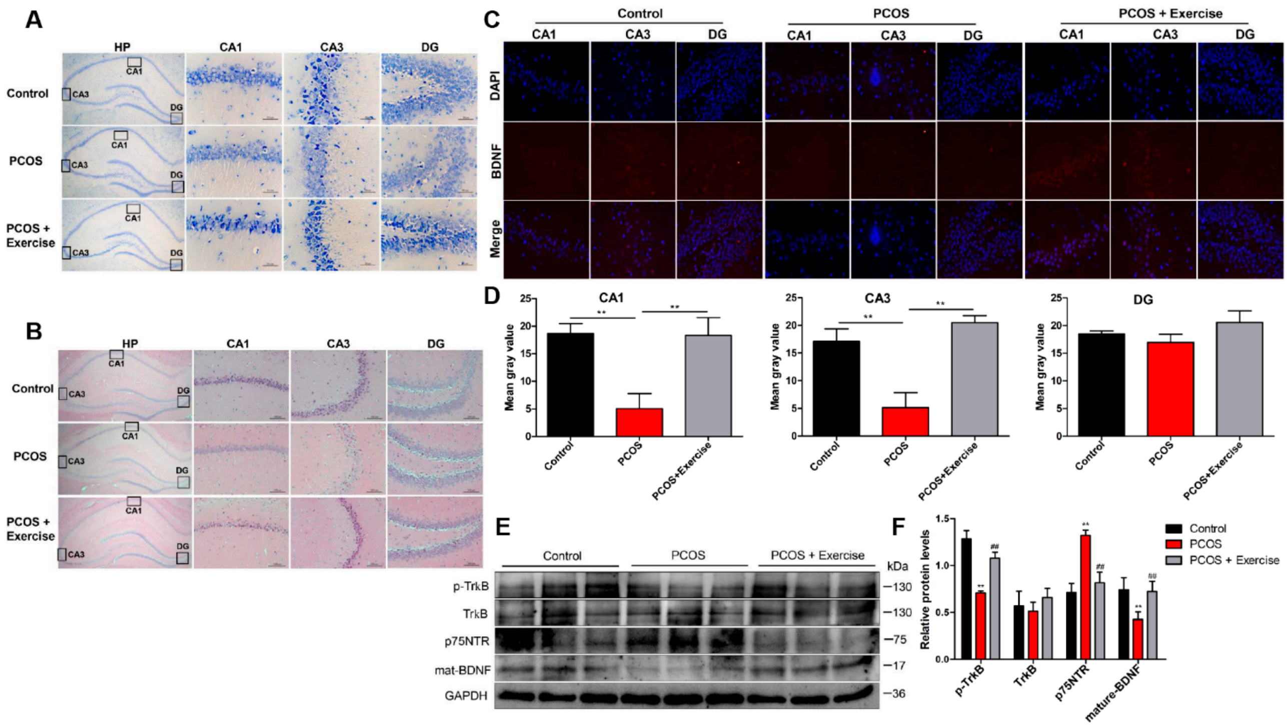

Figure S1: Aerobic exercise increased the expression of BDNF and its receptor in the hippocampus. Rats received DHEA for the induction of polycystic ovarian syndrome, together with or without exercise treatment. (A-B) Hippocampal neurons were assessed by Nissl's staining (40×) and H&E staining (20×). (C-D) The level of BDNF in CA1, CA3 and DG of hippocampal was measured with immunohistochemical staining. (E-F) The expression of BDNF, TrkB, and p75NTR in Hippocampal was assessed by western blot assay. n=10 in each group. Data are shown as mean ± SEM. **$P < 0.05$, vs. control group; ##$P < 0.01$, vs. PCOS group.

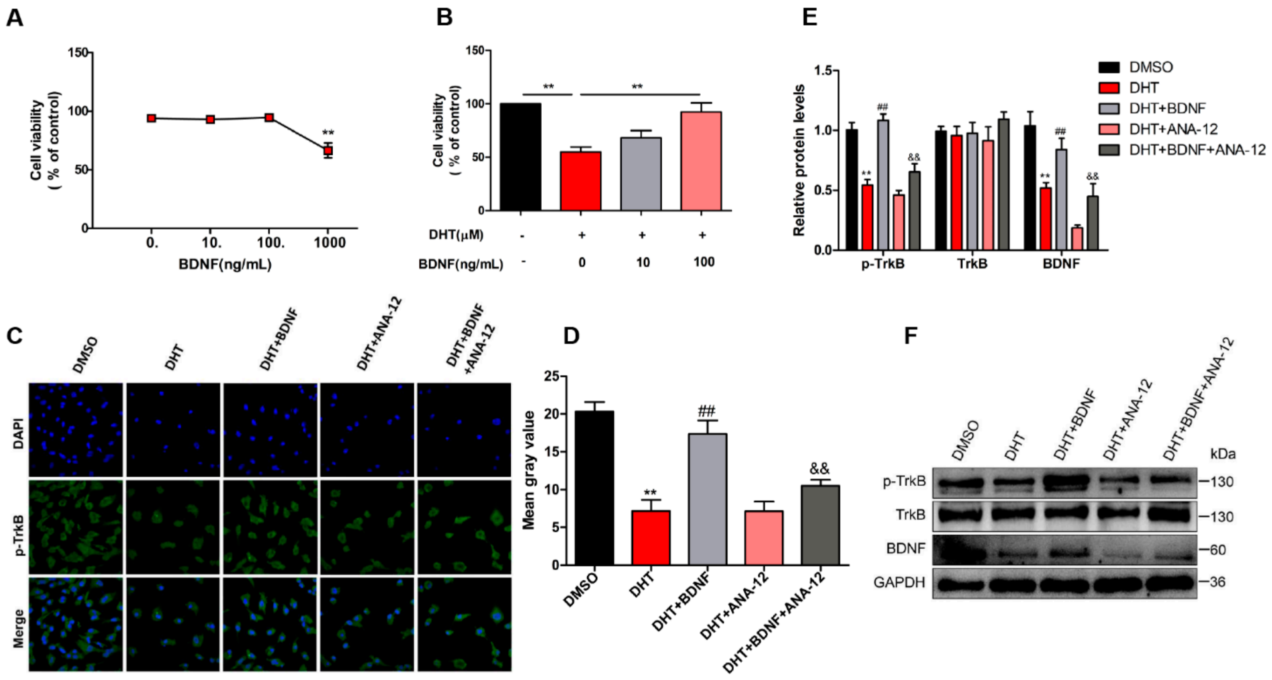

Figure S2: BDNF treatment increased the cell viability of DHT-induced GCs and provoked p-TrkB activation. Primary GCs were treated with BDNF and ANA-12 with or without DHT. (A-B) Cell viability of GCs after tunicamycin-induced was analyzed by CCK-8 kits. (C-D) Images of confocal laser scanning microscopy acquisitions of immunofluorescence staining of p-TrkB conjugate with Alexa Fluor™ 488 goat anti-rabbit IgG (H + L) in GCs treated with BDNF and ANA-12 (60×). (E-F) The expression level of TrkB, p-TrkB, and BDNF were detected by western blot assay. Data are shown as mean ± SEM. ***P* < 0.05, vs. DMSO group; ##*P* < 0.01, vs. DHT group; &&*P* < 0.01, vs. DHT + BDNF group.
